# Supplementary material for: Transport Properties of Methyl-Terminated Germanane Microcrystallites
Source: Nanomaterials (Basel). 2022 Mar 29;12(7):1128. doi: 10.3390/nano12071128 (PMC9000464; doi:10.3390/nano12071128)
Supplement: Supplementary file 1 [file nanomaterials-12-01128-s001.zip › nanomaterials-1533984-supplementary.pdf]

nanomaterials

Supplementary Material

Transport Properties of Methyl-Terminated Germanane Microcrystallites

Davide Sciacca [1], Maxime Berthe [1], Bradley J. Ryan [2], Nemanja Peric [1], Dominique Deresmes [1], Louis Biadala [1], Christophe Boyaval [1], Ahmed Addad [3], Ophélie Lancry [4], Raghda Makarem [4], Sébastien Legendre [4], Didier Hocrelle [4], Matthew G. Panthani [2], Geoffroy Prévot [5], Emmanuel Lhuillier [5], Pascale Diener [1] and Bruno Grandidier [1,*]

[1] Université de Lille, CNRS, Centrale Lille, Université Polytechnique Hauts-de-France, Junia-ISEN, UMR 8520-IEMN, F-59000 Lille, France; daveshake93@gmail.com (D.S.); maxime.berthe@iemn.fr (M.B.); nemanja.peric@iemn.fr (N.P.); dominique.deresmes@iemn.fr (D.D.); louis.biadala@iemn.fr (L.B.); christophe.boyaval@iemn.fr (C.B.); pascale.diener@junia.com (P.D.)
[2] Department of Chemical and Biological Engineering, Iowa State University, Ames, IA 50011, USA; bryan@iastate.edu (B.J.R.); panthani@iastate.edu (M.G.P.)
[3] Université de Lille, CNRS, INRAE, Centrale Lille, UMR 8207–UMET-Unité Matériaux et Transformations, F-59000 Lille, France; ahmed.addad@univ-lille.fr
[4] HORIBA FRANCE SAS, 455 Avenue Eugène Avinée 59120 Loos/Avenue de la Vauve–Passage Jobin Yvon, 91120 Palaiseau, France; ophelie.lancry@horiba.com (O.L.); raghda.makarem@horiba.com (R.M.); sebastien.legendre@horiba.com (S.L.); didier.hocrelle@horiba.com (D.H.)
[5] Institut des NanoSciences de Paris, CNRS, Université de Sorbonne, 4 Place Jussieu, 75005 Paris, France; geoffroy.prevot@insp.jussieu.fr (G.P.); lhuillier@insp.jussieu.fr (E.L.)
* Correspondence: bruno.grandidider@univ-lille.fr

1. Structural Characterization of the Microcrystallites

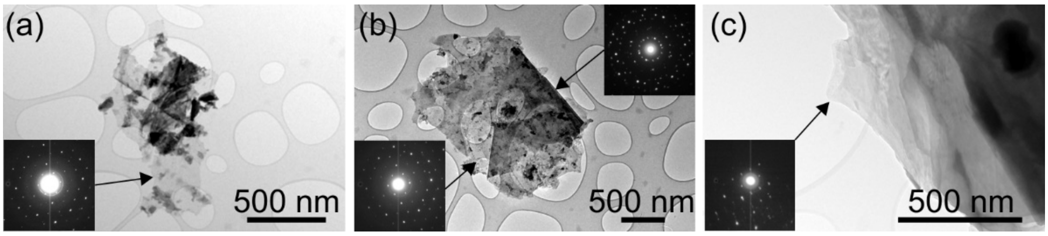

Figure S1. Room temperature structural analysis of (a,b) two flakes and (c) the edge of a microcrystallite drop-cast on a TEM grid after being stored in isopropanol for 4 years. The TEM images and selective area electron diffraction (SAED) patterns (insets) were acquired with a FEI Tecnai G2 transmission electron microscope operated at 200kV. While all the SAED patterns show a hexagonal structure consistent with germanane, multiple diffraction spots can be observed within flakes where the multilayers have different orientations (lower inset in (b)). The arrows point to the area where the SAED pattern were acquired.

2. Determination of the Microcrystallite Thickness

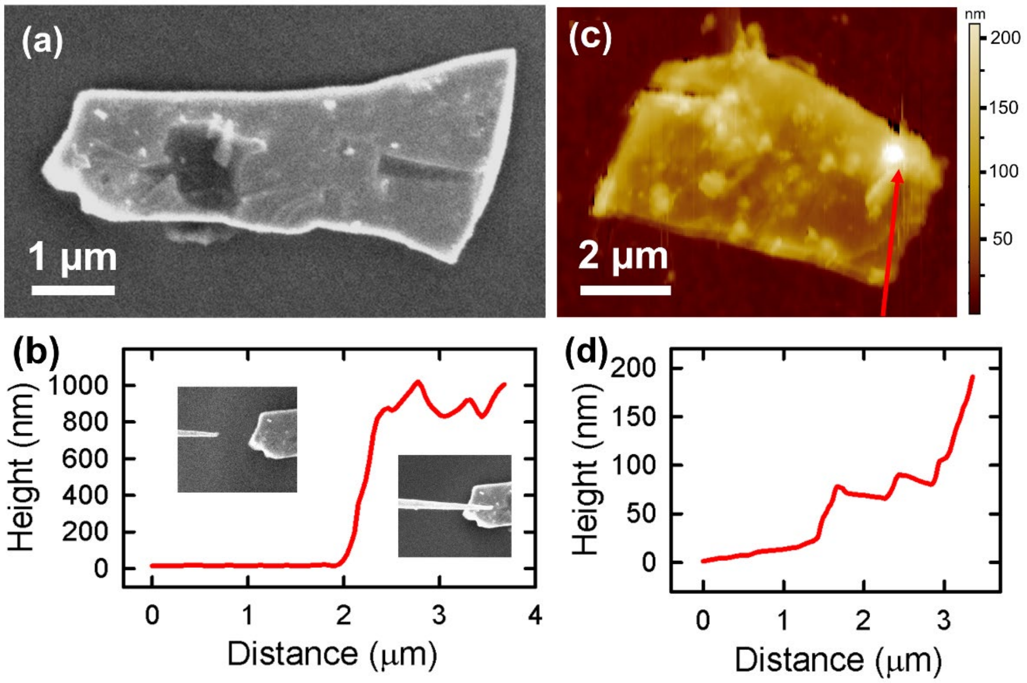

Figure S2. **(a)** Scanning electron microscopy (SEM) images of a methyl-terminated germanane microcrystallite annealed in ultrahigh vacuum. **(b)** Height profile measured with scanning tunneling microscopy (STM) with insets showing the STM tip either in contact with the Si substrate or in contact with the top left part of the microcrystallite seen in (a). Feedback conditions: sample bias: -3.0 V, tunneling current: 50 pA. **(c)** Atomic force microscopy image of a microcrystallite measured in tapping mode with a Dimension 3100 (Bruker) working in air. The height variation is provided by the color scale bar. The arrow indicates the position of the height profile shown in **(d)**. This height profile highlights the layered structure of the microcrystallite.

3. Influence of the Electrical Contact on the *V*(*I*) Characteristic

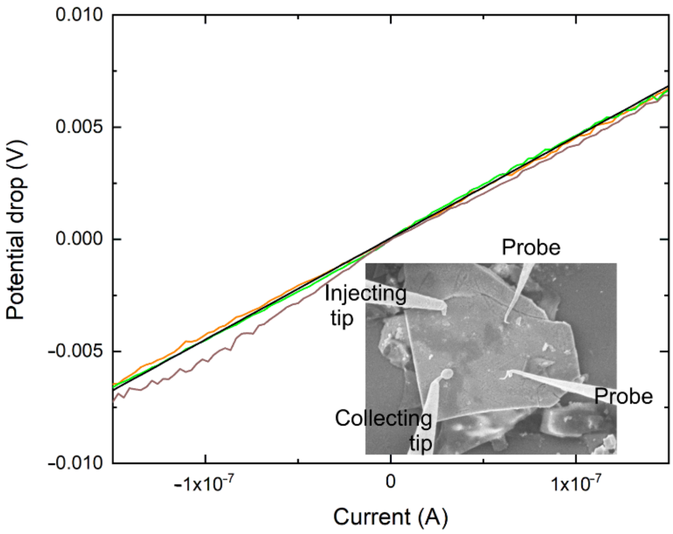

Figure S3. Influence of the strength of the electrical contact between the injecting tip and the surface of the microcrystallite on the *V*(*I*) characteristics. Three measurements were performed with a square arrangement of the tips. Between each measurement, the injecting tip was retracted and connected again. The black line corresponds to the best linear fit of the green curve and is used to highlight deviations from a straight line.

4. Additional Example of SEM Image Acquired at Different Length Scales

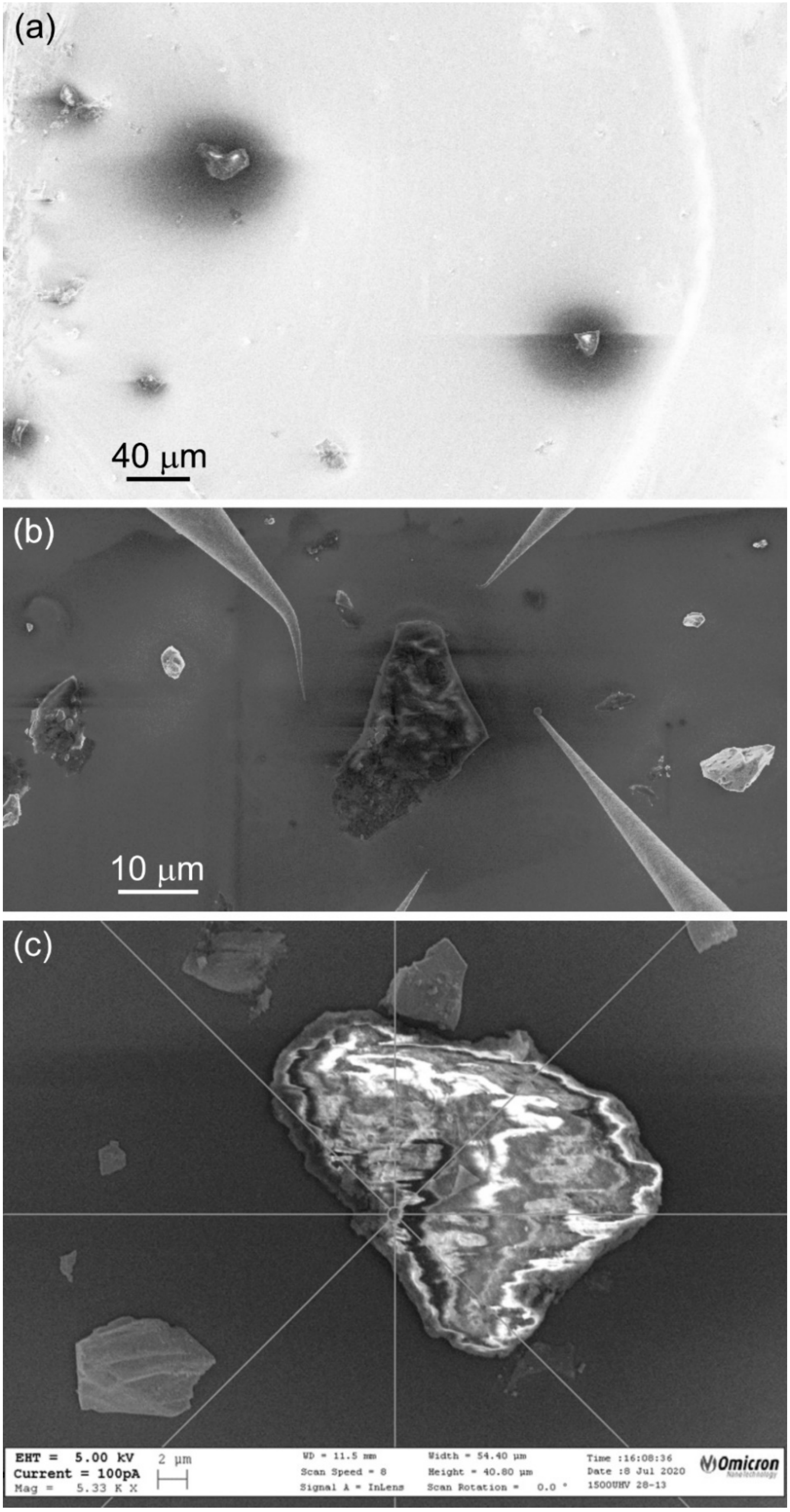

Figure S4. Three examples of areas imaged with scanning electron microscopy at increasing magnification showing that the largest microcrystallites are more affected by charging effects under the electron beam than the smaller microcrystallites. The accelerating voltage and probe current were set at 5 kV and 100 pA respectively.

5. Optical Characterization of the Microcrystallites in Air.

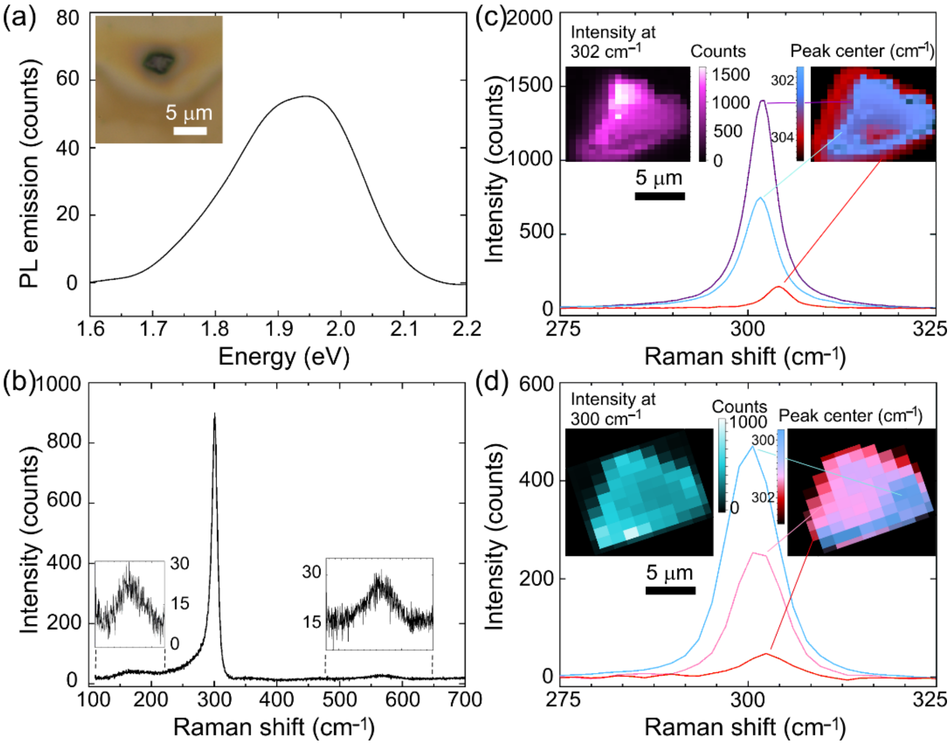

Figure S5. **(a)** Photoluminescence and **(b)** Raman spectroscopy acquired in the center of a methyl-terminated microcrystallite seen in the optical micrograph of the upper inset of panel (a). The small peaks measured in the Raman spectrum are magnified in the lower insets. **(c)** and **(d)** Spatially-resolved Raman spectroscopy of the same germanane microcrystallite measured a few hours after the microcrystallite was exposed to air and three weeks after the microcrystallite was left in ambient conditions, respectively. The intensity map in the left inset of panels (c) and (d) have been obtained at the wavenumber of 301.9 cm^{-1} and 300.6 cm^{-1}, respectively. The density maps in the right inset of panels (c) and (d) highlight the spatial dependence of the Raman E_{2g} mode. The maps consist of 20 × 20 and 10 × 10 pixels in (c) and (d) respectively. These experiments were performed in air with a LabRAM HR Evolution Raman microscope (Horiba Scientific) using a 532 nm laser excitation (100 mW), with respectively 1800 gr/mm and 300 gr/mm diffraction gratings and a 100x objective (0.9 NA). The Raman spectra were calibrated by setting the silicon phonon mode at 520.5 cm^{-1}.

6. Jump of a Microcrystallite due to Electrostatic Repulsion

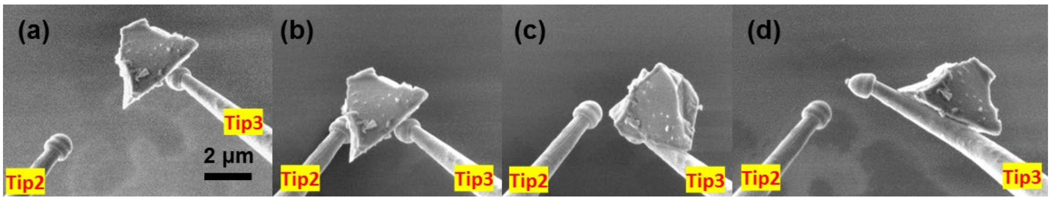

Figure S6. Sequence of SEM images showing the sudden jump of a microcrystallite that is caused by electrostatic repulsion. (a) Tip 3 is grounded and in mechanical contact with the microcrystallite. Tip 2 is polarized at +2.0 V. (b) When tip 2 is approached from the bottom of the microcrystallite to attempt a back contact, (f) the microcrystallite suddenly jumps onto Tip 3. Moving Tip 2 vertically results in a sliding motion of the microcrystallite along Tip 3. The accelerating voltage and probe current were set at 5 kV and 100 pA respectively.
